# Parenting Influences on Frontal Lobe Gray Matter and Preterm Toddlers’ Problem-Solving Skills

**DOI:** 10.3390/children11020206

**Published:** 2024-02-06

**Authors:** Josselyn S. Muñoz, Megan E. Giles, Kelly A. Vaughn, Ying Wang, Susan H. Landry, Johanna R. Bick, Dana M. DeMaster

**Affiliations:** 1 Department of Cognitive Sciences, Rice University, Houston, TX 77005, USA; josselyn.munoz@bcm.edu; 2 Children’s Learning Institute, The University of Texas Health Science Center at Houston, Houston, TX 77030, USA; megan.e.giles@uth.tmc.edu (M.E.G.); kelly.a.vaughn@uth.tmc.edu (K.A.V.); ying.wang@uth.tmc.edu (Y.W.); susan.landry@uth.tmc.edu (S.H.L.); 3 Psychology Department, University of Houston, Houston, TX 77204, USA; jrbick@uh.edu

**Keywords:** prematurity, neurodevelopment, neuroimaging, parenting, emotion regulation, cognition, frontal lobe, gray matter volume

## Abstract

Children born preterm often face challenges with self-regulation during toddlerhood. This study examined the relationship between prematurity, supportive parent behaviors, frontal lobe gray matter volume (GMV), and emotion regulation (ER) among toddlers during a parent-assisted, increasingly complex problem-solving task, validated for this age range. Data were collected from preterm toddlers (*n* = 57) ages 15–30 months corrected for prematurity and their primary caregivers. MRI data were collected during toddlers’ natural sleep. The sample contained three gestational groups: 22–27 weeks (extremely preterm; EPT), 28–33 weeks (very preterm; VPT), and 34–36 weeks (late preterm; LPT). Older toddlers became more compliant as the Tool Task increased in difficulty, but this pattern varied by gestational group. Engagement was highest for LPT toddlers, for older toddlers, and for the easiest task condition. Parents did not differentiate their support depending on task difficulty or their child’s age or gestational group. Older children had greater frontal lobe GMV, and for EPT toddlers only, more parent support was related to larger right frontal lobe GMV. We found that parent support had the greatest impact on high birth risk (≤27 gestational weeks) toddler brain development, thus early parent interventions may normalize preterm child neurodevelopment and have lasting impacts.

## 1. Introduction

Medical advances have improved the survival rate and physical needs of very preterm infants (VPT; less than 33 gestational weeks), but children born preterm continue to have elevated risk for neurodevelopmental difficulties (e.g., executive function, emotion regulation, language, etc.) [1,2,3,4]. These observed neurodevelopmental difficulties may result from brain injury at birth and/or disruption in utero brain development [5,6,7,8,9]. Early experiences, including duration and medical care in the neonatal intensive care unit (NICU) and parenting behaviors may also influence neurodevelopmental outcomes following preterm birth [10,11,12]. 

Parents play an important role in neurodevelopmental outcomes for children born preterm. Providing warm and contingent caregiving, which responds to the child’s attentional cues and meets their needs, has been associated with improved social–emotional and cognitive functioning in preterm-born children [13,14]. In addition, there is evidence that parental responsiveness directly influences the development of prefrontal gray matter with strong implications for improving self-regulation in children born VPT. For example, higher levels of parental sensitivity in early childhood are associated with larger total brain volume, as well as gray matter volume at 8 years [15]. Similarly, parental responsiveness may lead to better amygdala and prefrontal gray matter neurodevelopment, with the ultimate effect of reducing the risk of emotion regulation (ER) disruption and psychopathology among children. Yet, less is known regarding the effects of parental responsiveness on frontal limbic development and regulatory skills in children born preterm [16]. Taken together, the studies emphasize the importance of warm and responsive caregiving in optimizing neurodevelopmental outcomes for children, particularly those born preterm.

Early childhood is a crucial period marked by significant cognitive development, which serves as a foundational element for more nuanced cognitive functions later in life [17]. Self-regulation, or the ability to regulate one’s behavior in relation to what is environmentally appropriate, is contingent on executive functioning (EF) and emotion regulation (ER) [18]. Skills that prove vital for self-regulation are shaped during the first two years of life, and the emergence of these skills is intimately linked to development of prefrontal circuits [19,20]. Specifically, the prefrontal–limbic system, including frontostriatal connections, rapidly develops in the first three postnatal years. There is growing evidence that early self-regulatory dysfunction puts preterm children at increased risk for school failure and special education needs, as shown by teacher reports of behavioral and general academic delays [21,22,23]. 

Neuroimaging studies consistently link premature birth, particularly occurring before 33 weeks of gestation, with significant atypical white and gray matter microstructure [24]. Even at their term-corrected age, preterm infants exhibit reduced regional brain volumes. The prefrontal cortices that facilitate EF and ER skills encompass various frontal brain regions such as the anterior cingulate, as well as the medial, dorsal, and ventral prefrontal cortex [25]. Consistent with the idea that neural circuitry is most responsive to experience during rapid development, the protracted developmental course of the prefrontal cortex causes these networks to be heavily influenced by experience, and early in life, experiences will primarily stem from caregiver interaction [26,27,28,29,30,31,32,33]. Therefore, EF and ER typically develop in the context of relationships with caregivers and are facilitated by establishing a sense of safety and security, forming secure attachments, and coregulating emotions [34,35,36,37]. 

Within the context of prematurity, NICU stay length and clinical practices are an important environmental factor that shape developmental outcomes. Prolonged NICU stays have been associated with lower scores on the Bayley mental and motor scales during toddlerhood [11]. Interestingly, in this study, gestational age was not associated with the Bayley scales, suggesting that the severity of postnatal illnesses and NICU hospitalization may account for negative neurodevelopmental effects in already high-risk preterm infants. Increasingly, modifiable clinical care factors that may be at play include nutrition and sensory exposure, which are highlighted as targets for improved developmental outcome [12,38]. Moreover, limited parent–infant bonding during extended NICU stays may interfere with formation of secure attachment relationship. 

In order to measure and operationalize toddler ER, a task must include challenging elements that require the child to regulate their emotions often in the context of problem solving. The Tool Task was designed to measure aspects of early childhood problem-solving, decision-making, spatial awareness, and emotion regulation, as the child works on increasingly difficult tasks to remove toys from apparatuses with parent support [39]. This task was originally used to evaluate two-year-olds’ capacity to engage in ER, sustained attention, and problem-solving behaviors. Importantly, the Tool Task captures the toddler behavior within the context of increasingly complex challenges and measures the child’s ability to draw upon personal and environmental resources, such as their caregiver. This task also evaluates parenting behaviors as the extent to which caregivers provide supportive presence and quality of assistance as they work to help the child solve the task on their own. 

Consistent with this idea, children classified as having a secure attachment to their primary caregivers showed more enthusiasm, positive affect, persistence, cooperation, flexibility, resourcefulness, and engagement than insecurely attached children. Moreover, higher supportive caregiver behaviors during the task predicted lower negative child behaviors, including frustration, negative affect, and noncompliance behaviors [39]. During Tool Task levels, parental support and responsive behaviors are essential components of parent–child interactions, assessed by the scales of Supportive Presence and Quality of Assistance. These behaviors underscore a caregiver’s ability to provide support and assistance to a child during problem-solving situations, contributing significantly to the child’s positive and enjoyable learning experiences.

Supportive presence involves the parent’s attentiveness to the child and task, coupled with emotional responsiveness to the child’s signals. This creates a secure base for exploration, which is achieved by the parent staying calm, setting a positive mood, and being physically present. For example, the parent approaches the tool with obvious interest and enthusiasm. The parent makes certain that the child realizes there is a problem to be solved and indicates to the child that working on the problem can be rewarding. The parent may also indicate to the child that they are available to work cooperatively with him/her if it becomes necessary but encourages initial autonomous work to help the child achieve a sense of solving the problem her/himself. These supportive behaviors not only motivate but also reassure the child, leading to a high score on the Supportive Presence scale.

The current study examined the consequences of prematurity on frontal lobe gray matter volume (GMV) and emotion regulation (ER) among toddlers engaged in the Tool Task. This study tested associations between parent and child behaviors, with a special emphasis on toddler emotion regulation and parental support facilitated through coregulation, and toddler frontal lobe neurodevelopment for toddlers with varying levels of birth risk (i.e., extremely preterm, EPT; very preterm, VPT; and late preterm, LPT). The following hypotheses were tested: (1) We hypothesized that toddler ER during the Tool Task would vary based on interactive influences of birth risk, task difficulty, and toddler age. Specifically, we expected that there would be differences in ER between EPT, VPT, and LPT toddlers. (2) In terms of frontal lobe GMV, we anticipated that LPT toddlers would have the greatest frontal lobe GMV and EPT toddlers would have the least frontal lobe GMV. (3) Finally, we hypothesized that parent support would be most beneficial for EPT toddlers, both in terms of child behavior (e.g., more supportive parent behaviors associated with more toddler compliance and engagement) and in terms of frontal lobe GMV (e.g., more supportive parent behaviors associated with greater frontal lobe GMV).

## 2. Materials and Methods

This study included toddlers born preterm between 15–30 corrected gestational months and their primary caregivers. Sample demographics (*n* = 57) are presented in Table 1. The research team recruited the participants from two large pediatric clinics affiliated with the University of Texas Health Science Center located at the Texas Medical Center in Houston, TX, USA. Participants included in this sample had completed the initial testing: MRI and Tool Task. MRI data were successfully collected from 35 participants. This study is part of a larger longitudinal study that utilizes scalable parenting interventions to test if parental responsiveness is a modifiable psychological factor that improves neurodevelopmental outcomes and brain connectivity in toddlers born preterm. Tool Task data were collected in a laboratory setting at the Children’s Learning Institute in Houston, TX, as part of baseline assessment, and was administered along with other parent reports and behavioral measures for the larger study. Behavioral assessments were video recorded and coded (see Supplementary Materials for coding framework). MRI data were acquired, concurrently with behavioral data, at Baylor College of Medicine Core for Advanced Magnetic Resonance Imaging in Houston, TX. The study participants received a gift card for completing this behavioral testing session. Prior to any testing, informed consent was obtained from all parents involved in this study; children enrolled in this study were too young to provide assent for participation. 

**Figure 1:**
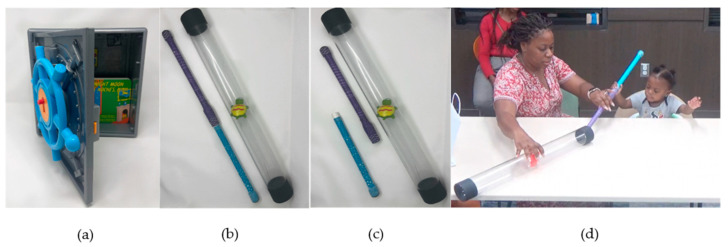
Tool Task apparatus levels with parent–child interaction. (**a**) Vault toy apparatus corresponding to difficulty Level 1. (**b**) Short tube apparatus corresponding to difficulty level 2. (**c**) Long tube apparatus corresponding to difficulty level 3. (**d**) Mother supporting child well in level 2 of the Tool Task. Notice the parent’s hand-over-hand assistance and child’s active engagement in the task.

The study procedure was approved by the Committee for the Protection of Human Subjects at the University of Texas Health Science Center at Houston. The Tool Task was administered according to standard guidelines [39], optimized for our study design. Each child was seated in a booster seat next to their parent at a table. The three levels of the Tool Task increased in difficulty, increasing the need for emotion regulation and parent assistance while problem-solving (Figure 1a–d). During a 1 to 1.5-h baseline testing session, the Tool Task was the first task administered. The Tool Task had three levels that took between one to four minutes to complete and that increased in difficulty. The first level, “vault toy,” asked the child to turn a wheel until the vault unlocked to release the prize (a book) that was inside (Figure 1a). The second level was the “short tube” task, in which the child inserted a stick inside a clear tube to retrieve the toy stuck in the middle (Figure 1b). The third level, the “long tube” task, was similar to the second level (Figure 1c). However, for the third level, the parent and toddler needed to realize that they were not able to release the toy stuck in the middle of the clear tube by using one stick, instead they had to put together both halves of the sticks so that it was long enough to push the toy through the tube. The parent was present for all problem-solving tasks and instructed to let the child try to solve the problem independently, but they were able to provide as much help as they thought their child needed (Figure 1d). These tasks, especially the third level, tended to be stressful, thus requiring child emotion regulation and increased coregulation from parent to child.

Behavioral coding measures and framework. For each level, three coders analyzed six variables for child behaviors and two variables for caregiver behaviors (interrater reliability, α = 0.95). Each of the videos had 24 total ratings across the child and caregiver variables (8 variables per 3 levels). Coders began analyzing behaviors when the examiner put the toy and apparatus on the table and said, “Can you get the toy out of the box or tube?” Coders stopped coding approximately ten seconds after the child retrieved the prize. The only coding exclusion was when the parent was talking to the examiner or other adults in the room. Behavioral coding materials are included in Supplementary Materials.

Child behavioral measures. Child *noncompliance* was measured on a 1 to 6 scale evaluating the extent to which the child attended to the caregiver and complied with caregiver requests. A high score would mean more noncompliance as shown by the child refusing all caregiver offers of support and never following caregiver directions. A low score, in contrast, means that the child attended to most of the parent’s requests and followed the instructions. Child *engagement* was measured on a scale of 1–7 and was defined as the degree to which the child is interested, engaged in, and enthusiastic about the task. A score of 1 reflects the child’s active effort to avoid the task, whereas a score of 7 reflects very high levels of engagement and thorough involvement in the task, and the middle of the scale reflects moderate levels of engagement. Other child behaviors did not have enough variability to examine (see Table 2).

Caregiver behavioral measures. Coders observed the supportive presence and quality of assistance from the caregiver. Using a 1 to 7 scale, coders rated the emotional support with which the parent helped the child have a positive learning experience. Higher ratings meant that the parent met most criteria and subcriteria (providing secure base, attentiveness to child, helping their child focus, reinforcing, staying calm, anticipating frustration, setting a learning and enjoyable mood, etc.). The quality of assistance measure also used a 1 to 7 scale to evaluate the sensitivity with which the caregiver maximized the child’s learning opportunities. If the caregiver received the highest score, then they were excellent at giving assistance. To show the combination of warmth and contingent responsiveness, we summed the two parent variables in the analysis for an overview of their level of assistance provided (i.e., Parent Assistance + Parent Supportive Presence = Parent Assistance Sum).

Toddler MRI Scans. MRI data were collected within seven days on average after the behavioral data (36% on the same day) during the toddlers’ natural sleep. T1 and T2 weighted images were collected using a 64-channel head coil on a Siemens 3T scanner. The parents were instructed to perform their usual bedtime routine with the child and to alert the research staff once the child had been asleep for fifteen minutes. Then, researchers transferred the child from the bed into the scanner. Hearing protection included earplugs, pediatric earmuffs, and a thick piece of foam curved around the interior of the bore to act similar to an acoustic hood [40]. A member of the research team was inside the scanner, monitoring the child for movement and distress. If the child awoke, the scan was stopped immediately. 

**Figure 2:**
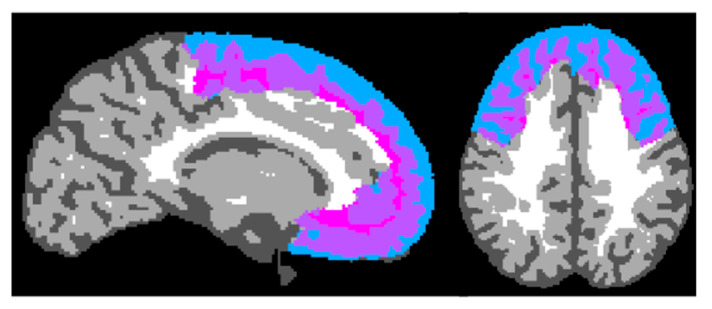
iBEAT brain segmentation example. The blue represents cerebrospinal fluid, the purple is gray matter, and the pink is white matter.

The T1 and T2 scans were processed using Infant Brain Extraction and Analysis Toolbox (iBEAT V2.0 Cloud) for initial processing and brain segmentation [41,42,43,44,45]. The neuroimaging processing steps included the following: heterogeneity correction, skull stripping, and tissue segmentation. FSL was then used for gray matter volume computation to calculate total gray matter volume and gray matter volume within each parcellation of the UNC-BCP 4D Infant Brain Volumetric Atlas (Figure 2) [46]. All frontal regions included in analyses are presented in Table 3. There was no significant difference between the right and left frontal gray matter volumes (*t*(34) = 1.94, *p* = 0.06).

Statistical Analysis. We conducted linear mixed effects models with task difficulty (levels 1, 2, and 3) as a within-subjects variable and gestational group (EPT, VPT, and LPT) and age (adjusted for prematurity) as between-subjects variables. We used these models to predict toddler and parent behavior during the Tool Task. To examine brain structure differences across gestational groups, we conducted linear mixed effects models with hemisphere (right/left) as a within-subjects variable and gestational group (EPT, VPT, and LPT) and age (adjusted for prematurity) as between-subjects variables, controlling for total GMV. We also explored the relationships between parent behaviors during the Tool Task, child behaviors during the Tool Task, and bilateral frontal lobe GMV. A power sensitivity analysis was performed using G*Power version 3.1 [47]. With a sample size (N) of 57 and three gestational groups, the study demonstrates a relative effect size ranging from 0.423 to 0.546, ensuring a statistical power (1-β err prob) of 0.80 and 0.95 [48].

## 3. Results

Descriptive statistics for all Tool Task outcome variables by gestational group are presented in Table 2, with age included as a covariate. Birth risk was unrelated to parent assistance and toddler noncompliance, anger, coping, and persistence during the Tool Task. There was a significant difference in toddler engagement across the gestational groups: in all three levels of the Tool Task, LPT toddlers were more engaged than VPT or EPT toddlers. 

### 3.1. Toddler Noncompliance

**Figure 3:**
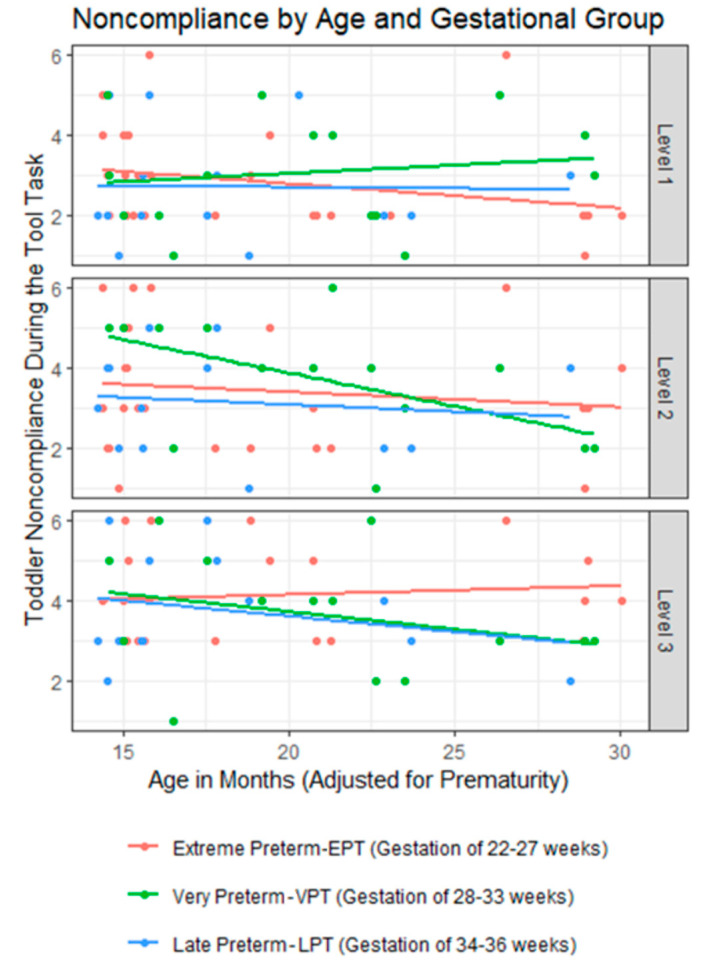
Toddler noncompliance by levels (1–3) of the Tool Task and three gestational groups.

There was a significant three-way interaction between gestational group, task difficulty, and adjusted age (*F*(4,88) = 3.23, *p* = 0.02). The pattern generally reflects increased compliance for older toddlers as the task increased in difficulty, but this relationship differed by gestational group (see Figure 3). For LPT toddlers, who had the lowest birth risk, older toddlers were more compliant than younger toddlers at level 3 of the Tool Task only; there was no relationship between compliance and age for levels 1 and 2. For VPT toddlers, who had moderate birth risk, older toddlers were more compliant than younger toddlers at levels 2 and 3 of the Tool Task; there was no relationship between compliance and age for level 1. For EPT toddlers, who had the highest birth risk, older toddlers were more compliant than younger toddlers at level 1 of the Tool Task only; there was no relationship between compliance and age for levels 2 and 3. 

### 3.2. Toddler Engagement

The three-way interaction between gestational group, task difficulty, and adjusted age was not significant for toddler engagement during the Tool Task (*F*(4,88) = 2.035, *p* = 0.10). There was a significant main effect of task difficulty (*F*(2,88) = 3.84, *p* = 0.03) and a significant main effect of adjusted age (*F*(1,44) = 5.04, *p* = 0.03). Engagement decreased with task difficulty and increased with age. 

### 3.3. Parent Support

Parents did not differentiate their support during the Tool Task depending on their child’s age (*F*(1,46) = 2.43, *p* = 0.13), their child’s gestational group (*F*(2,46) = 1.93, *p =* 0.16), or task difficulty (*F*(2,45) = 1.52, *p* = 0.22).

### 3.4. Parent Support and Toddler Noncompliance

Because parent support did not differ by task difficulty, we created a sum score to represent overall parent support across all three levels of the task (see Table 2). In a model predicting toddler noncompliance from parent support, gestational group, and age, toddler noncompliance was not related to overall parent support (*F*(1,45) = 2.33, *p* = 0.13).

### 3.5. Parent Support and Toddler Engagement

In a model predicting toddler engagement from parent support, gestational group, and age, there was significant interaction between parent support and gestational group (*F*(2,43) = 5.23, *p =* 0.01). To better understand this interaction, exploratory univariate analyses were conducted for each gestational group, testing main effects of parent support on child engagement within each level, such that effect of parenting support at each task level was tested relative to child behavior at the same task level. Univariate results indicated that EPT toddlers who received more parent support were more engaged (*F*(1,22) = 6.75, *p* = 0.02) at level 2; whereas, for LPT toddlers parental support predicted child engagement at level 3 (*F*(1,10) *= 8.39, p* = 0.02). Parent support did not predict child engagement for VPT toddlers. Of note, a summed score of parent support was evaluated in the primary analysis, which was appropriate for testing differences in child behavior across levels. However, to evaluate child behavior within each task level, a level-specific score for parent support was deemed more suitable. 

### 3.6. Frontal Lobe GMV

Frontal lobe GMV was unrelated to the gestational group (*F*(2,28) = 0.60, *p* = 0.62). Across gestational groups, older children had greater frontal lobe GMV than younger children (*F*(1,28) = 12.24, *p* = 0.002). 

### 3.7. Frontal Lobe GMV and Parent Support

**Figure 4:**
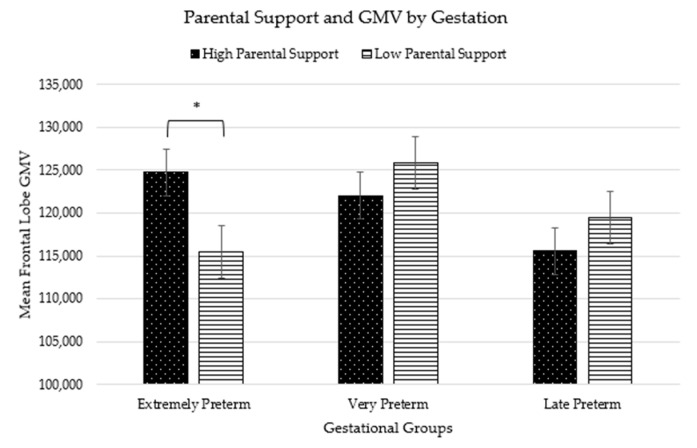
Graph of parental support and GMV by gestation shows the significant effect of high parental support on EPT child frontal GMV. * *p* < 0.05.

At levels 2 and 3, there was a significant interaction between gestational group and parent support in predicting right frontal lobe GMV (level 2: *F*(3,22) = 4.27, *p* = 0.01; level 3: *F*(3,21) = 6.17, *p =* 0.004). The interaction indicated that parent support was related to right frontal lobe GMV only for EPT toddlers; EPT toddlers with parents who were more supportive during the Tool Task had greater right frontal lobe GMV than EPT toddlers with parents who were less supportive during the Tool Task (see Figure 4). Parent support was unrelated to frontal lobe GMV for VPT and LPT toddlers. There were no significant interactions between gestational group and parent support for the left frontal lobe GMV. 

### 3.8. NICU Stay Length

The current study analyzed the duration of NICU stay in days and its potential impact on GMV and ER. Results showed a non-significant effect of NICU stay duration on total GMV or frontal GMV (*Fs*(1,34) ≤ 0.35, *ps* ≥ 0.56). The effect of NICU stay duration on toddler ER throughout the three levels of the Tool Task was also nonsignificant (*Fs*(1,53) ≤ 3.45, *ps* ≥ 0.07).

## 4. Discussion

Previous research indicates that utilizing brain plasticity during the critical period of toddlerhood can serve as a mechanism to promote healthy developmental outcomes for preterm (PT) children [34]. Responsive interactions with caregivers have the potential to enhance neurodevelopmental outcomes, including cognition, language, and brain microstructure, for children at risk of cognitive, psychiatric, and behavioral disorders, particularly those born extremely premature [33]. Both healthy toddlers and those born preterm are at a pivotal stage in neurodevelopment, wherein environmental stimuli, especially parenting, must be adaptively responsive to address each child’s evolving needs. This adaptability is crucial for fostering positive neurodevelopmental outcomes. The reciprocal interaction between parents and toddlers during play, as well as routine activities such as grocery shopping and dressing, provides the necessary stimulation for the healthy development of brain regions associated with emerging language, cognitive skills, and emotion regulation capacities. Taking a step further, actively supporting brain development, particularly in the frontal lobes, through responsive parenting could be a key factor in improving neurodevelopmental outcomes among extremely preterm individuals, who frequently encounter challenges related to frontal lobe processes such as executive function (EF) and emotion regulation (ER). 

This study makes a significant contribution to the existing literature by illustrating the positive impact of responsive parenting on brain development, leading to increased gray matter volume (GMV) in the frontal lobe of extremely preterm toddlers. Supporting our hypothesis, older toddlers were more compliant as the Tool Task became harder. One possibility is that, across gestational groups, older toddlers are better able work as a team with their caregivers to solve difficult problems—that is to say, older toddlers are better able inhibit their own agenda to make use of caregiver supports and suggestions. Regarding gestational effects, LPT toddlers are more compliant only in the third level, but at moderate level difficulty older VPT toddlers were more compliant at levels 2 and 3. EPT toddlers showed a similar pattern, but only at level 1, the easiest level of the task. Regardless of age, EPT toddlers were moderately noncompliant throughout levels 2 and 3. This pattern reflects the perception of difficulty for each gestational group, as EPT toddlers find it difficult at level 1, VPT toddlers find it difficult at level 2, and LPT toddlers find it difficult at level 3. This effect may be due to the lower birth risk allowing for typical developmental patterns to become evident at this age.

Supporting our hypothesis, child engagement decreased as the task became more challenging, but increased with child age. It is likely that the frustration inherent to increased task difficulty leading led to lower engagement, although older children were better able to regulate negative emotions and allocate attention to achieving the goal, leading to higher engagement across difficulty levels. Like the age-related effects, LPT toddlers exhibited the highest levels of engagement across all task levels, possibly owing to their ability to employ emotion regulation, enabling them to focus on the task goals. Exploratory analysis suggests that parent support is an individual difference; thus, the absence of differences in parent support may be attributed to consistent personality traits rather than the state of assisting the child during problem-solving.

Whereas parent support was not related to toddler noncompliance, it did impact toddler engagement. LPT and EPT toddlers with supportive parents demonstrated higher engagement in the task. Importantly, the effects of parent support on child engagement were noticeable at level 3 for LPT toddlers and level 2 for EPT toddlers, underscoring the need for parental support to be adaptively tailored to meet the changing needs of toddlers. The observed patterns of child engagement and parent support in challenging tasks align with the concept of scaffolding in Vygotsky’s sociocultural theory. Within the caregiving context, Vygotsky’s scaffolding principal centers on providing support and guidance to a learner, adjusting the level of assistance as needed. EPT and LPT toddlers, possibly due to their specific developmental readiness, may benefit from more effective parental scaffolding at level 2 and level 3, respectively, facilitating their engagement and success at an appropriate point in this challenging task [49,50].

Frontal lobe GMV only differed by age, where older children had greater GMV than younger children due to increased neurodevelopment as they age. The differences by age are similar across the three groups as expected. In line with our hypothesis that parent support benefits high birth risk toddlers the most, there was an interaction between gestational group and parent support, such that the high birth risk group (EPT) showed greater right frontal lobe GMV with greater parent support. It is unclear why parenting effects were right lateralized, one possibility is that right frontal lobe may be more involved in ER and thus more malleable to environment specific stimulation, such as parenting behavior [51]; whereas left frontal lobe may be more sensitive to other environmental factors, such as rich language exposure. Although the constrained sample size of this study warrants caution, it is essential to consider the finding that parental behavior impacted the frontal lobe GMV in the gestational group at the highest neurodevelopmental risk (i.e., EPT). This finding aligns with the differential susceptibility theoretical framework [52], which suggests that individuals more susceptible to adversity may also be more responsive to positive influences, such as supportive parenting. Within the context of prematurity, according to this framework, those at the highest neurodevelopmental risk may benefit the most from supportive parenting practices. Consistent with our hypothesis, EPT toddlers who received substantial parental support during the task exhibited significantly greater gray matter volume in the frontal lobe. This EPT group experienced the most atypical developmental journey and longest postnatal period, distinguishing them from the later gestational groups. In this cohort with the highest birth risk, parental support appears to play a crucial role in mitigating GMV deficiencies among preterm children, potentially contributing to improved cognitive and socioemotional development and lowering the risk of psychiatric disorders. Subsequent research, including a larger sample and longitudinal design, should delve into the effects of parental intervention on preterm toddler GMV, emotion regulation, and the intricate interplay with parental support.

Present findings highlighting the significant influence of parent support on the brain development of toddlers at the highest birth risk are compelling but should be considered within the context of the following limitations. First, to achieve these study aims, multiple hypotheses were tested on a relatively small sample size, increasing the risk for false positives. Toddlers born with low gestational ages (EPT and VPT) constitute a specialized population, which increases the potential impact of research findings; yet researchers working *with* pediatric patient populations and their families are aware of health-related (e.g., ongoing outpatient therapies, respiratory illness) barriers for recruitment and enrollment, ultimately limiting sample size. For this reason, the modest sample size in this study necessitates cautious interpretation of results. Future studies with larger samples are recommended to enhance generalizability and robustness of findings. Furthermore, the cross-sectional nature of this study does not capture developmental change over time. Instead, our study design allows for the inference of age-related changes in both brain and behavior, alongside current environmental experience. Future longitudinal studies are essential to provide a more comprehensive understanding of the dynamic developmental and even infer differential trajectories of brain–behavior outcomes in preterm toddlers.

The inclusion of MRI data in this research provides valuable insights into the intricate relationship between brain development and behavioral outcomes in toddlers born preterm. Although the acquisition of MRI data from sleeping toddlers represents an innovative approach, it is important to note that a limitation was that frontal lobe volumetric analyses were restricted to toddlers who remained asleep during MRI data acquisition. This limitation might introduce bias, as our sample could be skewed toward toddlers who experience less disruption during natural sleep. An anecdotal note of interest—investigators in this study collect several variables on child sleep habits. Surprisingly, sensitivity to sound during sleep and regular bedtime are not sufficient predictors of success; whereas, multiple MRI data collection attempts and same day behavioral and MRI testing support MRI data acquisition. One final limitation, the study did not assess motor skill ability or milestones, and it is plausible that EPT and VPT toddlers faced additional challenges in this domain compared to LPT toddlers. While the Tool Task’s coding framework allows for scaling of coded behaviors based on child capacity, future investigations should consider incorporating fine motor skill assessments to provide a more comprehensive understanding of the interplay between parenting behaviors, emotion regulation, and motor development challenges in preterm infants and toddlers.

## 5. Conclusions

Based on our results that parent support has the greatest impact on the highest birth risk (under 28 weeks gestation) toddler brain development, parent interventions may be warranted to normalize their child’s brain development from the start and create a lasting positive impact. Future work research should target increasing positive parenting behavior to improve brain development, ER, and other cognitive skills in toddlers born preterm. Continuing to investigate the effects of parenting interventions on preterm toddlers could establish the way for this population to have better ER/EF mechanisms and reach their full developmental potential.

## Figures and Tables

**Table 1:** Descriptive characteristics of participants (*n =* 57).

Sample Characteristics	n (%)
Child Sex	
Male	30 (52.63)
Female	27 (47.37)
Child Race	
Black or African American	20 (35.09)
White	21 (36.84)
Asian	2 (3.51)
American Indian or Alaska Native	1 (1.75)
Native Hawaiian or Other Pacific Islander	1 (1.75)
Declined to respond	12
Child Ethnicity	
Yes, Hispanic or Latino	30 (52.63)
No, not Hispanic or Latino	24 (42.10)
Declined to respond	3
Gestation Classification	
Extreme Preterm (22–27)	28 (44.92)
Very Preterm (28–33)	15 (26.32)
Late Preterm (34–36)	14 (24.56)
Caregiver Relationship to Child	
Mother	52 (91.2)
Father	4 (7)
Other	1 (1.8)
Caregiver Education: Highest Grade Completed	
Primary school, Finished 5th grade	2 (3.5)
Middle School	4 (7)
Some High School	8 (14)
High School diploma or GED	7 (12.3)
Vocational or technical training	2 (3.5)
Some College	11 (19.3)
Bachelor’s degree (BA/BS)	15 (26.3)
Master’s degree MA, MS, JD	4 (7)
Other	3 (5.3)
Declined to respond	1 (1.8)
	Means (SD)
Adjusted Gestational Age in months	19.24 (4.904)
Gestational Age at Birth (weeks)	28.60 (4.39)
Primary Caregiver Age	32.74 (7.22)

**Table 2:** Gestational group differences in behavioral measures of the Tool Task using ANCOVA, with age as a covariate.

Measure	Extremely Preterm	Very Preterm	Late Preterm	*F(df)*	*p*
	Means (SE)	Means (SE)	Means (SE)		
Child Behavioral Measures					
Child Noncompliance					
Level 1	2.824 (0.277)	2.946 (0.360)	2.523 (0.373)	0.350 (2,46)	0.707
Level 2	3.210 (0.294)	3.795 (0.382)	3.080 (0.395)	1.005 (2,46)	0.374
Level 3	4.127 (0.281)	3.682 (0.365)	3.734 (0.377)	0.608 (2,46)	0.549
Child Anger ^+^					
Level 1	1.044 (0.193)	1.421 (0.251)	1.392 (0.260)	0.954 (2,46)	0.393
Level 2	1.083 (0.198)	1.470 (0.257)	1.886 (0.266)	3.012 (2,46)	0.059
Level 3	1.564 (0.292)	1.373 (0.380)	2.140 (0.393)	1.065 (2,46)	0.353
Child Coping ^+^					
Level 1	2.869 (0.157)	3.220 (0.204)	3.380 (0.211)	2.158 (2,46)	0.127
Level 2	2.781 (0.178)	3.015 (0.231)	3.140 (0.239)	0.809 (2,46)	0.452
Level 3	2.607 (0.172)	2.946 (0.223)	2.907 (0.231)	0.945 (2,46)	0.396
Child Engagement					
Level 1	3.921 (0.259)	3.776 (0.337)	5.151 (0.349)	5.018 (2,46)	0.011 *
Level 2	4.010 (0.299)	3.394 (0.388)	4.865 (0.401)	3.418 (2,46)	0.041 *
Level 3	3.191 (0.299)	3.539 (0.388)	4.620 (0.401)	4.145 (2,46)	0.022 *
Child Persistence ^+^					
Level 1	3.176 (0.241)	3.193 (0.313)	4.019 (0.324)	2.455 (2,46)	0.097
Level 2	3.225 (0.266)	2.634 (0.346)	3.612 (0.357)	1.941 (2,46)	0.155
Level 3	2.660 (0.249)	2.846 (0.323)	3.383 (0.335)	1.520 (2,46)	0.229
Parent Assistance Sum					
Level 1	10.500 (0.615)	9.911 (0.799)	11.672 (0.827)	1.195 (2,46)	0.312
Level 2	11.017 (0.527)	9.888 (0.685)	11.705 (0.708)	1.720 (2,46)	0.190
Level 3	10.271 (0.522)	10.109 (0.678)	12.173 (0.702)	2.884 (2,46)	0.066
Across Levels				3.84 (2,46)	0.029 *

^+^ not hypothesized or included in analyses; * *p* < 0.05.

**Table 3:** Gestational group differences in frontal gray matter using ANCOVA, with age and total gray matter volume as covariates.

Measure	Extremely Preterm	Very Preterm	Late Preterm	*F(df)*	*p*
	Means (SE)	Means (SE)	Means (SE)		
Left Frontal Gray Matter Regional Volumes					
Sum of frontal regions	58,678 (1056)	58,346 (1427)	60,719 (1354)	0.911 (2,30)	0.413
Middle frontal gyrus	10,261 (367)	10,743 (497)	10,803 (471)	0.533 (2,30)	0.592
Precentral gyrus	7228 (236)	7197 (319)	7691 (303)	0.867 (2,30)	0.430
Supplementary motor area	4172 (169)	4137 (229)	4342 (217)	0.255 (2,30)	0.776
Medial orbitofrontal cortex	1506 (107)	1421 (144)	1424 (137)	0.165 (2,30)	0.849
Inf. orbitofrontal cortex	4181 (223)	4306 (301)	5003 (286)	2.684 (2,30)	0.085
Middle orbitofrontal cortex	1915 (123)	1580 (166)	2161 (158)	3.147 (2,30)	0.057
Medial sup. frontal gyrus	5093 (279)	4920 (377)	4914 (358)	0.107 (2,30)	0.898
Dorsal sup. frontal gyrus	5455 (230)	5581 (311)	5439 (296)	0.067 (2,30)	0.935
Rolandic operculum	3649 (71)	3513 (96)	3738 (91)	1.436 (2,30)	0.254
Triangular inf. frontal gyrus	6521 (171)	6226 (231)	6788 (219)	1.522 (2,30)	0.235
Opercular inf. frontal gyrus	2450 (69)	2452 (93)	2594 (88)	0.930 (2,30)	0.405
Rectus gyrus	1792 (198)	2029 (268)	1365 (254)	1.662 (2,30)	0.207
Anterior cingulate cortex	4455 (147)	4243 (199)	4457 (189)	0.422 (2,30)	0.660
Right Frontal Gray Matter Regional Volumes					
Sum of frontal regions	59,944 (1460)	59,620 (1973)	63,532 (1973)	1.386 (2,30)	0.266
Middle frontal gyrus	10,241 (486)	10,323 (657)	10,950 (623)	0.427 (2,30)	0.656
Precentral gyrus	7219 (307)	7291 (416)	7849 (394)	0.844 (2,30)	0.440
Supplementary motor area	4356 (175)	4748 (236)	4762 (224)	1.411 (2,30)	0.260
Medial orbitofrontal cortex	2159 (113)	2075 (152)	2259 (144)	0.379 (2,30)	0.688
Inf. orbitofrontal cortex	4638 (198)	4341 (267)	5202 (254)	2.824 (2,30)	0.075
Middle orbitofrontal cortex	2348 (163)	1958 (221)	2743 (209)	3.254 (2,30)	0.053
Medial sup. frontal gyrus	3252 (242)	3423 (327)	3253 (310)	0.100 (2,30)	0.905
Dorsal sup. frontal gyrus	6383 (301)	6266 (407)	6458 (386)	0.058 (2,30)	0.944
Rolandic operculum	4618 (75)	4386 (101)	4624 (96)	1.967 (2,30)	0.157
Triangular inf. frontal gyrus	4798 (116)	4538 (157)	5238 (149)	5.316 (2,30)	0.011 *
Opercular inf. frontal gyrus	3678 (123)	3768 (166)	3885 (158)	0.541 (2,30)	0.588
Rectus gyrus	1708 (191)	1864 (258)	1488 (245)	0.557 (2,30)	0.579
Anterior cingulate cortex	4546 (144)	4642 (195)	4822 (185)	0.686 (2,30)	0.511

* *p* < 0.05.

## Data Availability

Data are contained within the article and Supplementary Materials.

## Supplementary Materials

The following supporting information can be downloaded at: https://www.mdpi.com/article/10.3390/children11020206/s1.
