# Supplementary material for: Parenting Influences on Frontal Lobe Gray Matter and Preterm Toddlers’ Problem-Solving Skills
Source: Children (Basel). 2024 Feb 6;11(2):206. doi: 10.3390/children11020206 (PMC10887128; doi:10.3390/children11020206)
Supplement: Supplementary file 1 [file children-11-00206-s001.zip › children-2827101-supplementary.pdf]

Dyadic problem solving between parents and toddlers: Coding framework for the Tool Task

Ying Wang, Megan Giles, & Dana DeMaster

This research was funded by the National Institutes of Health (NIH), grant number R01HD100560.

Table of Contents:

Tool Task Overview

1. **Three levels:** The parent and child complete three problem solving tasks that are graded in difficulty. The assessor explains the problem to the parent and gives the tool and apparatus to the child. The parent is instructed to allow the child to solve the problem independently, but provide as much help as they believe the child needs. Note this tends to be a mildly stressful task that also requires child emotion regulation.
   - A. **Vault toy:** Level 1 is the "vault toy" task, in which the child must turn a wheel until the vault unlocks in order to release the prize (book) from inside.
   - B. **Short tube:** Level 2 is the "short tube" task, in which the child must insert a stick inside a clear tube to get a target toy.
   - C. **Long tube:** Level 3 is the "long tube" task, which requires the child to put two short sticks together then use it in the same manner as level 2 to get the toy out of a longer tube. The child must realize they cannot reach the toy with the half sticks given to them, and the only way to get the toy out is by putting the two halves together and pushing it through the tube.

2. Units of analysis:

- A. **Ratings are for each level:** There are six variables for the child behaviors, and there are 2 variables for the caregiver behaviors for each of the three levels. In total, there are 24 ratings per video.

Child Scales	Caregiver Scales
Noncompliance (1-6)	Supportive Presence (1-7)
Anger (1-6)	Quality of Assistance (1-7)
Anger/frustration toward caregiver (1-7)	
Coping (1-5)	
Persistence (1-5)	

Engagement (1-7)	

B. **Start and stop points:** The starting point is when the examiner puts the toy and tool on the table and says “Can you get the toy out of the box/tube?”. The stopping point is 10 seconds after the child gets the prize out.
C. **Behaviors excluded from coding:** The only behavior excluded from coding is if the parent is talking to the examiner or other adult in the room.

Child Scales Overview

For each of the three tasks, this manual outlines four emotional regulation variables and two attention variables for the child. The emotional regulation variables include noncompliance, anger, anger/frustration toward caregiver, and coping. The attention variables include persistence and enthusiasm for the task.

Noncompliance

Definition: This scale measures the degree to which the child shows willingness to listen to caregiver's suggestions in the setting and to comply with their requests in a reasonable manner. The scale also evaluates the child’s enthusiasm and persistence in the task.

Child Noncompliance Scale		
At the low end, a child matches his behavior to maternal directions in a detailed fashion, e.g., if the caregiver asks the child to turn the wheel the child turns the wheel. The child also is attentive to the caregiver and may focus his activity on her directions to the extent that she provides direction.	1	Child actively orients toward caregiver's directions in the session and complies to all major task instructions plus most details about specific behaviors on the tasks. Thus, the child molds his behavior into a collaborative effort with caregiver on the tasks, heeding her suggestions with a compliance that suggests a basic trust in her advice and direction and acceptance of her authority as a guide in this situation. The child may not do everything the caregiver suggests, but these behaviors reflect autonomy within a

		compliant orientation rather than intentional negativism.
	2	Child complies with most major directions of the caregiver, e.g., staying on task or returning to task efforts at her direction, accepting her ideas on how to do the tasks. He/She may occasionally not comply, but, generally, when caregiver repeats or gets firmer, compliance follows.
At intermediate scale points, the child shows a mixture of compliant and rejecting responses to caregiver's plans, acts as though incognizant of caregiver's suggestions either because the child is involved in his own schedule of activity or the caregiver gives few directions with which to comply.	3	The child basically seems generally compliant toward the caregiver's demands and willing to work in collaboration with her, but the child's own schedule of activities sometimes leads to noncompliance. Child does not seem invested in rejecting the caregiver's directions, and episodes of noncompliance are brief and followed by behavior indicating acceptance of caregiver's leadership.
	4	There are major, but isolated, episodes of noncompliance during the session, or tendency toward noncompliance throughout, that make the interaction difficult and strained. Yet the child does comply eventually in most instances except after becoming upset.
At the high end of the scale, the child actively refuses to comply with maternal directions throughout most of the session. The child may do so by overt denial of her demands and	5	Child shows a strong tendency toward noncompliance, but it is mixed with a few efforts to follow suggestions and directions given by the caregiver. Noncompliance is more sporadic and probably patterned to frustrating and difficult moments of the session compared to the above level but is still strong.

pulling away from the caregiver or leaving the task, rejecting her physical efforts to help solve the task, and acting contrary to her suggestions.	6	Child rejects virtually all directions of the caregiver during the session. Early in the session and continuing throughout, the child refuses to obey the caregiver. Commands and suggestions may be followed at initial steps but are regularly sequenced with refusals to comply. In effect, the child does little demanded of him and demonstrates almost a total negativism toward the caregiver.

Anger

Definition: This scale refers to the degree of anger expressed by the child, regardless of its origin or locus. The child may be involved in angry interaction with the caregiver, or may kick at the tools, throw objects, etc. Expression of anger towards the parent can be double-coded under "Anger" and "Anger/Frustration Directed to Caregiver."

Child Anger Scale	
1	No signs of anger.
2	An isolated sign or two; for example, jerking on the sticks or one forceful "No!" to the caregiver or a vigorous shake of the head.
3	Isolated signs or one brief angry period, from which the child rather quickly recovers, or angry signs only after a long period of frustration or difficulty, or longer periods of subtle resistance.
4	More than one angry period or signs of anger of some intensity throughout.
5	Several angry periods or one full-blown tantrum.
6	More than one tantrum or an angry tone which predominates throughout, or one full-blown tantrum that persists for a minute or more.

Anger/Frustration directed toward Caregiver

Definition: Degree to which the child shows anger, dislike, or hostility specifically directed toward the caregiver.

Child Anger/Frustration directed toward Caregiver Scale		
At the low end, there are neither overt nor covert signs of such anger. Expressions are essentially positive toward the caregiver whether or not the child is compliant or much involved with her.	1	Child shows no signs of anger or frustration directed at the caregiver. He/She shows through consistently positive interactions toward the caregiver that she/he has a truly positive relationship toward her and feels no abiding anger toward her, or he/she may ignore her. The child may show frustration, but this is directed at the tool or elsewhere, not at the caregiver.
	2	Child shows no clear indications of negativism, but the tone of some interactions is less positive than one would desire in an ideal relationship toward the caregiver.
	3	Child is angry only briefly in any overt fashion, but these suggest some noticeable anger and resistance in the child's interactions with the caregiver.
	4	Child shows clear frustration/anger toward the caregiver on several occasions, but these are rather isolated episodes separated by periods in which the child behaves quite positively toward the caregiver or pays little attention to her.
At the high end, the child is repeatedly and overtly angry at her, e.g., forcefully rejecting her ideas, showing angry and resistant	5	Child frequently directs frustration toward the caregiver or shows very strong frustration toward the caregiver. These are not predominant in the interaction, but very much disrupt other activities when these episodes occur.

expressions, pouting, or being unreasonably demanding of her.	6	Child's anger is a predominant aspect of their interactions, but it is shown in more sporadic and generally subtler ways than above.
	7	Child is repeatedly and overtly angry, resistant, or frustrated toward the caregiver. The degree of anger here seems so strong that the child cannot disguise it in subtler ways for long, but it repeatedly appears in his interactions with her. It is a (or the) dominant theme in the session.

Coping Behaviors

Definition: Coping behaviors pertain specifically to the degree of stress and frustration the child can tolerate and observation of coping or self-soothing behaviors. That is, the child may look fine until the situation gets taxing. If deterioration happens immediately at that point, a very low coping score would result.

Child Coping Scale	
1	Extremely low coping ability. It is little exaggeration to say that this child finds entering the room too stressful. Even with support this child becomes disorganized in the face of challenge. He/She may not be able to even engage Tool 3 (or even 2), and once frustrated, the child cannot get back together.
2	Little ability to cope with frustration. Only when things are going well can this child stay organized. Anything beyond moderate challenge leads to disorganization. If the caregiver is very supportive, such a child can be nursed through the tools.
3	This child has reasonable coping abilities for a two-year-old. He/She can tolerate some frustration and is not disorganized by challenge, but limits to frustration tolerance are clearly seen. Tool 3 is likely beyond this child's capacity though he/she may start out well. (Tool 2 could lead to disorganization if things went quite poorly.)
4	Child handles considerable frustration or challenge before becoming

	disorganized, but limits may be seen (or the task goes so smoothly that no frustration is involved).
5	The child stays organized and involved in the face of great challenge or frustration.

Persistence

Definition: The degree to which the child remains goal-oriented and involved with the problem. Poor support or assistance on the part of the caregiver may interfere with the child's efforts. An attempt is made to keep this independent of maternal behavior, i.e., the child's persistence is viewed in the context of the caregiver's behavior. A high score means that this child is remarkably persistent or persistent to a remarkable degree given the lack of support experienced.

Child Persistence Scale	
1	Little persistence - quickly disengages or never really tries. Cannot be engaged except for brief periods despite suitable efforts by the caregiver.
2	Periods of engagement and disengagement are mixed. Perhaps the child shows the pattern on both tools or perhaps he withdraws quickly on tool 3 even though it went smoothly on tool 2.
3	Average persistence. This child will show some signs of fading or decreasing even without severe frustration, or cannot be induced to re-engage at all following mild frustration or a lengthy period of non-success. Must show some sustained engagement for a time.
4	Notable persistence. This child is quite persistent in the face of less than full support or completely persistent in the context of strong support, i.e., he is not given sufficient opportunity to show his own inner staying power. Leaving the task, becoming disorganized, refusing to try further or failure to stay involved may well occur following considerable frustration.
5	Child remains goal-oriented and involved almost no matter what. Keep in mind that the most persistent two-year-old can be beat if the

	odds are great enough. Eventually, any child will give up or fall apart.

Engagement

Definition: The degree to which the child is interested, engaged in, and enthusiastic about the task.

Child Engagement Scale	
1	Active effort to avoid the task. The child may initially be interested in the task but quickly tires and tries to leave the situation.
2	Little enthusiasm for the task and not very engaged. The child works only in response to the caregiver's requests or directives. The child may leave the task, but is directed back by the caregiver. Even then he requires prodding to continue his efforts.
3	The child is working at the task but only in response to a considerable amount of encouragement on the caregiver's part. The child does at times muster some enthusiasm on his own, but seems quite dependent on the caregiver for motivation.
4	The child is trying hard for the major portion of the task, but is rather passive at times, perhaps requiring some encouragement on the part of the caregiver to keep working, or perhaps leaving the task very briefly and quickly returning on his own to the task at hand. The child would be characterized as moderately interested, but not enthusiastic about the task.
5	Going about the task with interest.
6	Trying hard and showing high levels of engagement, but not as vigorous as the activity involved in a rating of 7.
7	Very engaged, thoroughly involved in the task, and obviously enjoying the activity. Involvement must be with respect to the task rather than to some non-task behavior. Child gives the impression of working so hard that he would not want to be dragged away.

Caregiver Rating Scales

Supportive Presence

Definition: The emotional support with which the parent helps the child have a positive and enjoyable learning experience. This category includes the following major criteria and subcriteria:

Major criteria

1) Secure Base – helping the child feel comfortable with the task
2) Caregiver Involvement – attentiveness to child and task

Subcriteria (components of major criteria)

a) Focusing the child on the task when needed
b) Tuning the child in to reinforcing aspects of the task as needed
c) Mood setting for a problem solving situation as needed
d) Helping the child achieve a sense of having solved the problem him/herself
e) Sharing in the joy of solution
f) Encouraging and supporting his/her efforts
g) Physical presence when needed
h) Anticipating frustration and taking action to help the situation
i) Caregiver staying calm

Caregiver Supportive Presence Scale	
1	All criteria and subcriteria are not met, or there is a distinct negative quality to the interaction. The mother reacts to the child's performance by becoming angry, hostile, cold, and/or totally unavailable.
2	Most subcriteria and both major criteria are not met. The caregiver's support is relatively absent, although there is no distinct negative quality characterizing the interaction.
3	The caregiver is lacking in areas of both major criteria and could be characterized as supportive in only a weak manner. They have some relative strengths and may be able to fulfill 2-4 subcriteria. It may be the case that one major criterion is *minimal* and at least *four* subcriteria are not met.
4	The caregiver is not non-supportive, but the degree of support is not obvious or striking.

5	The caregiver's presence has a positive effect but she is not as supportive and/or involved as in higher ratings. One major criterion may be minimally met.
6	The caregiver technically meets all criteria (if applicable) but is not quite able to give the child all the support required.
7	The caregiver meets all criteria and subcriteria. If the task is very easy for the child, he/she may not require as much support as indicated by the criteria.

Quality of Assistance

Definition: The skills and sensitivity with which the caregiver helps the child solve the problem and maximizes the child's chance to learn relationship rules and concepts. This category includes the following major criteria and subcriteria:

Major Criteria

1) Giving minimal assistance needed to keep the child working toward a solution
without solving it for him/her.
2) Helping the child see the relationships between actions that are required to
solve the problem.

Subcriteria (components of major criteria)

a) Grading of hints
b) Clarity of hints – working in tune with the child's level
c) Flexibility – changing instructions for more effective help
d) Timing of instructions
e) Pacing of instructions
f) Cooperating – giving hints that meet the child's needs
g) Having control of the situation
h) Giving space initially
i) Comments are helpful rather than discouraging
j) Effectiveness of instructions

Caregiver Quality of Assistance Scale	
1	The caregiver distracts the child, frustrates him/her, or simply provides no assistance.
2	**Very poor**. Nearly all or all subcriteria are unmet.
3	**Weak**. Neither major criterion is met, although they may be scored minimally.
4	**Moderately good to weak** at giving assistance to the child. They show more than a few strengths.
5	**Moderately good** at giving assistance. It may be that one major and one or two subcriteria are not met, or three subcriteria are not met.
6	**Quite good**. The two major criteria are met but perhaps not 100 percent, or all but two of the subcriteria are met.
7	**Excellent**. The caregiver meets all subcriteria and criteria or they are excellent at giving assistance. All major criteria and subcriteria apply, or both major and all but one subcriterion are met.

TOOL TASK Administration Script
Bilingual Spanish/English

Follow directions for each level.

- **Wait at least 10secs after the child gets the prize before moving on to the next level. The 10sec waiting period begins when the child grabs the prize, even if the child puts the prize back.**
- **The parent-child pair have 7 minutes to complete the level. If they do not complete the level in 7 minutes the task is over and the pair do not advance to the next level.**

Level 1:
You will need a book and the lockbox toy

To the parent: **"This is a task for your child to work on, but you can provide help if your child needs it"**
"Este es un juego para que su hijo trabaje, pero usted puede brindar ayuda si su hijo la necesita"

To the child: **"For this game, I'm going to hide a little prize inside a toy but I'm going to be tricky. Watch me."**
"Para este juego, voy a esconder un pequeño premio dentro de un juguete, pero voy a ser tramposo/a. Mírame".

- Start task with lockbox unlocked
- Place the book inside
- Lock the box, and say: **"It's locked now."** ***"Esta Cerrado"*** *while trying to open it*
- Say **"Can you get the toy out of the box?"** ***"¿Puedes sacar el juguete de la caja?"***

Levels 2-3:
The procedure is the same as above but with different tools. Levels 2 and level 3 will use a stick and a tube. Place the bath toy in the center of the tube before the session. Provide the

parent and child with the tools.
Level 2:
You will need a bath toy, the screw together stick (**screwed together**), and the **shorter** clear tube.

To the parent: **"Just like in the last game, this is a task for your child to work on, but you can provide help if your child needs it"**
"Al igual que en el último juego, este es un juego para que su hijo trabaje, pero usted puede brindar ayuda si su hijo la necesita"

To the child: **"Can you get the toy out of the tube?"**
"¿Puedes sacar el juguete del tubo?"
Level 3: Little bath toy
You will need a bath toy, the screw together stick (**unscrewed**), and the **longer** clear tub.

- Covertly mention to parent goal of task is to **screw the sticks back together**

To the parent: **"Just like in the last game, this is a task for your child to work on, but you can provide help if your child needs it"**
"Al igual que en el último juego, este es un juego para que su hijo trabaje, pero usted puede brindar ayuda si su hijo la necesita"

To the child: **"Can you get the toy out of the tube?"**
"¿Puedes sacar el juguete del tubo?"

Level 2:
Use Shorter tube
Paired sticks
Face the tube-hole in front of child showing the color stickers hanging down

Level 3:
Use Longer tube
Unpaired sticks
Face the tube-hole in front of child

RA instruction:
Both stickers and toys should be put at least half way into the paired tube
If child is too frustrated, do not proceed to next level
Remember to repeat instructions on each level!
Finishing RA will prepare tubes for the next assessor
In level 3:

- Covertly mention to parent goal of task is to screw the sticks back together
